# Acute Post-Match Fatigue and Recovery Kinetics of Neuromuscular Performance and Muscle Damage After a Friendly Match in Elite Youth Football Players

**DOI:** 10.3390/jfmk11030353

**Published:** 2026-09-03

**Authors:** David Bernatík, Karel Hulka, Andrea Izakova

**Affiliations:** 1 Department of Sport, Palacký University, 771 11 Olomouc, Czech Republic; david.bernatik01@upol.cz; 2 Faculty of Sports Science and Health, Matej Bel University, 974 01 Banská Bystrica, Slovakia; andrea.izakova@umb.sk

**Keywords:** repeated sprint ability, explosive strength, fatigue, muscle damage, recovery

## Abstract

**Background and Objectives**: The purpose of this study was to investigate the acute fatigue response to a standardized friendly football match in elite adolescent players and to describe the subsequent recovery of neuromuscular performance, biochemical markers, and subjective perceptions over a 72-h period. **Methods**: Thirty-seven elite male youth football players (16.3 ± 0.7 years) participated in this repeated-measures study. Neuromuscular function was assessed using the countermovement jump (CMJ) and repeated sprint ability (RSA) tests. Muscle damage was evaluated by measuring blood creatine kinase (CK) concentrations, while subjective recovery was monitored using a visual analog scale (VAS) for muscle soreness and the Well-Being Questionnaire (WBQ). Measurements were obtained before the match, immediately after the match, and again 24, 48, and 72 h later. **Results**: The friendly match induced significant neuromuscular, biochemical, and perceptual fatigue. CMJ height, reactive strength index, and repeated sprint performance decreased significantly immediately post-match (*p* < 0.001). Perceived muscle soreness increased markedly and remained elevated at 24 h. Neuromuscular performance largely recovered within 48 h, with 20-m sprint performance no longer significantly different from pre-match values at 48 h. In contrast, CK concentrations peaked at 24 h and remained significantly elevated throughout the 72-h recovery period. Significant correlations were observed between subjective soreness, CK concentrations, and neuromuscular performance during recovery. Recovery following match play differed depending on the marker used. Functional performance largely returned to baseline within 48 h, whereas CK concentrations remained elevated throughout the 72-h observation period. **Conclusions**: These findings emphasize the value of combining objective performance tests with subjective measures when monitoring post-match recovery in elite youth football players.

## 1. Introduction

Football match play imposes considerable physiological and neuromuscular demands on players, resulting in acute fatigue and a temporary reduction in physical performance [1]. These responses arise from a combination of factors, including depletion of muscle glycogen stores, dehydration, thermoregulatory stress, and exercise-induced disturbances in muscle metabolism that impair excitation–contraction coupling and force production [2,3].

The functional consequences of match-induced fatigue are commonly reflected in reduced sprinting and jumping performance. Field-based assessments such as the countermovement jump (CMJ) and repeated sprint ability (RSA) tests have therefore become widely used tools for evaluating neuromuscular recovery after competition [4,5,6]. At the same time, biochemical markers, particularly creatine kinase (CK), provide information about exercise-induced muscle damage [7,8], while perceptual measures including muscle soreness and self-reported well-being offer valuable insight into the athlete’s perceived recovery status [9].

Recovery after a football match is a complex process because physiological systems do not recover simultaneously. Neuromuscular function, biochemical responses, and subjective perceptions follow different recovery trajectories, and complete restoration may require up to 72 h depending on the marker being assessed [10]. Consequently, relying on a single indicator may not provide an accurate representation of an athlete’s readiness to resume training or competition.

Recovery responses in adolescent players differ from those reported in adults [11]. Young athletes generally demonstrate lower glycolytic capacity, reduced reliance on anaerobic metabolism, and distinct neuromuscular fatigue characteristics, all of which influence both the magnitude of fatigue and the subsequent recovery process [12,13]. Previous studies have suggested that youth players often experience less peripheral fatigue and recover more rapidly after high-intensity exercise, particularly during the pre- and early-pubertal years, when central mechanisms appear to contribute more substantially to fatigue development [14,15,16].

Despite growing interest in recovery monitoring, most available evidence has been obtained from adult football players. Comparatively few investigations have examined elite youth athletes using a comprehensive approach that combines neuromuscular, biochemical, and perceptual measures under standardized match conditions [17]. A more integrated understanding of post-match recovery in this population may help practitioners optimize training schedules and reduce the risk of excessive fatigue during congested competition periods.

Thus, the aim of this study was to investigate the post-match fatigue and recovery profile of elite adolescent football players following a friendly match. Specifically, the study aimed to (a) identify indicators of acute neuromuscular and metabolic fatigue, and (b) examine the recovery time course of these responses over a 72-h period.

## 2. Materials and Methods

### 2.1. Participants

Of the 40 players (without goalkeepers) enrolled in the study, 37 completed all required assessments across the measurement time points. Three players had incomplete observations due to illness (*n* = 1) or muscle injuries (*n* = 2) and were therefore excluded from the repeated-measures analyses (age = 16.3 ± 0.7 years; 2.4 ± 0.8 years post-PHV, with a predicted age at PHV of 13.9 ± 0.5 years; height = 171.8 ± 11.5 cm; body mass = 63.9 ± 9.1 kg). Players who competed in the highest competitive level with comparable performance in the Czech Republic volunteered to participate in this study. All players had at least seven years of football experience and trained a minimum of five times per week, in addition to regular weekend matches.

### 2.2. Procedures

**Figure 1:**
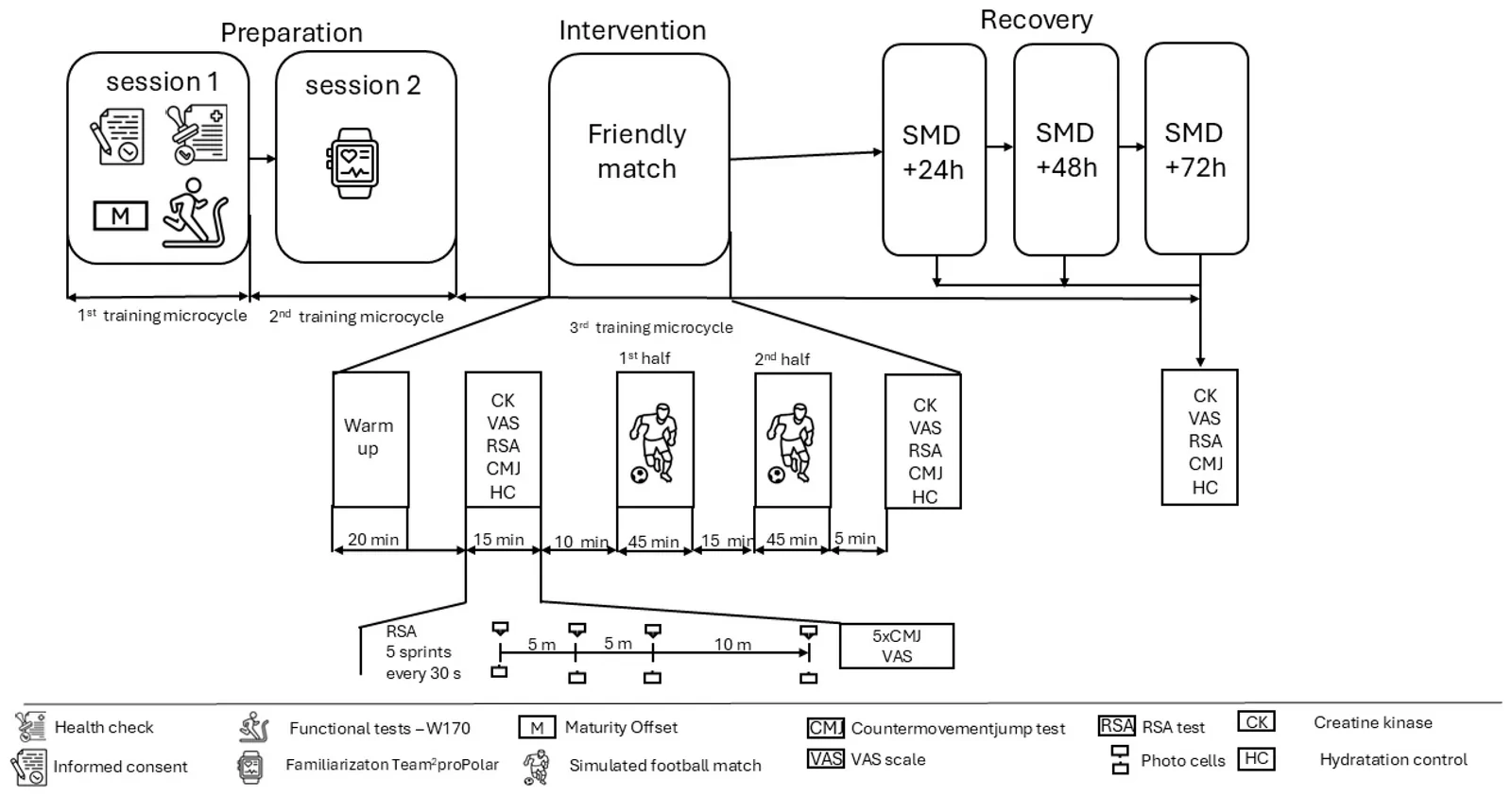
The design of the study.

This study used a prospective repeated-measures within-subject design to examine the recovery profile of elite adolescent football players following a standardized friendly match (Figure 1). All testing sessions were conducted on an outdoor football field over a two-week period. During the first and second microcycle (preparation phase), participants completed informed consent, health screening, anthropometric measures, estimation of biological maturation, and familiarisation with the Team^2^ Pro Polar monitoring system. They were also introduced to all performance assessments, including the CMJ test, RSA test, and VAS.

Measurements (intervention phase) were collected before the friendly match following the warm-up (pre–M), instead of the WBQ, which was administered in the morning. The next measurements were taken immediately after the match (post–M), and 24 h (M + 24), 48 h (M + 48), and 72 h (M + 72) after the match. These time points were selected to evaluate acute fatigue responses and recovery patterns over the following three days (recovery phase).

The assessment battery included blood creatine kinase concentration (CK), perceived well-being assessed using the Well-Being Questionnaire (WBQ) and expressed as the Hooper Index (HI), muscle soreness assessed using VAS, and neuromuscular performance evaluated through CMJ and RSA tests.

The first testing session was scheduled on a Monday afternoon to ensure a 72-h recovery period after the last training session. Before the match, all participants completed a standardized 20-min warm-up consisting of moderate-intensity running, static and dynamic stretching, and short acceleration runs. The friendly match followed official football rules and consisted of two 45-min halves played in an 11-versus-11 format without substitutions.

At M + 24, players completed the warm-up and all testing procedures. The M + 48 session included testing followed by a 60-min recovery-oriented training session. The final assessment (M + 72) was performed immediately after the warm-up and before a scheduled 90-min training session.

### 2.3. Data Collection

#### 2.3.1. Maturity Offset and Biological Age

Biological maturation was estimated using maturity offset (PHV) according to the anthropometric prediction equation for boys proposed by Mirvald et al. [18]. Standing height, sitting height, body mass, and chronological age were used to calculate maturity offset. Biological maturation was expressed as the predicted age at peak height velocity (PHV), calculated as chronological age minus maturity offset for each individual player.

#### 2.3.2. Hydration Control (HC)

Hydration status was additionally monitored through changes in body mass. Relative body mass loss (%) was calculated as: ∆BM (%) = ((BM_PRE_ − BM_POST_) × BM_PRE_^−1^) × 100. Values exceeding 2% were considered indicative of meaningful dehydration according to ACSM guidelines [19].

#### 2.3.3. Repeated Sprint Ability Measurement (RSA)

Participants completed a series of 5 × 20 m sprints (approximately 3 s) starting every 30 s with active recovery consisting of walking back to the start. Two seconds before each sprint, the participants were instructed to assume their starting positions [20,21]. The electronic timing gates (Brower Timing Systems, Draper, UT, USA) were pointed at the start line, the 5-m mark, the 10-m mark, and at the finish line at 20 m. All were set at a height of 0.70 m above the ground. Two scores for each distance were calculated: total sprint time (TT) as the sum of five sprints and the best sprint of the five sprints for each distance. Total sprint time demonstrated excellent reliability (CV = 1–4%; ICC > 0.90) [20,22].

#### 2.3.4. Countermovement Jump Test (CMJ) and VAS Scale

Participants performed five consecutive jumps to maximize jump height and minimize ground contact time [23]. All jumps were performed from an upright standing position with hands placed on the hips. The performance was assessed using the OptoJump system (Microgate S.R.L., Bolzano, Italy), a mobile optical measurement device.

Each participant completed two trials separated by a 3-min recovery period, and the best attempt was used for analysis. Average jump height and reactive strength index (RSI) were calculated. RSI was determined as the ratio between flight time and contact time (FT/CT). This CMJ protocol has previously shown good-to-excellent reliability for detecting small performance changes in elite players [23].

Perceptual responses were assessed using VAS for muscle soreness. Participants rated soreness in the lower limbs using a 100 mm line, where 0 mm represented “no soreness during repeated maximal jumps” and 100 mm represented “the greatest soreness ever experienced during repeated maximal jumps”. The VAS assessment was completed immediately after the CMJ test.

Previous research has reported acceptable reliability for post-exercise VAS measurements, with reliability coefficients ranging from 0.80 to 0.87 and a standard error of measurement (SEM) between 7.1% and 10% [24]. According to Kelly [25], the minimal clinically important difference for severe pain is approximately 10 mm (confidence interval: 6–14 mm).

#### 2.3.5. Level of CK Measurement

CK level was measured in capillary blood samples. A qualified professional performed sample collection. The procedure involved piercing the skin of the fingertip with a lancet (Accu-Chek, Roche Diabetes Care GmBH, Mannheim, Germany) and collecting blood samples using a pipette. The collected blood (32 μL) was applied to a magnetic test strip (Reflotron^®^CK strips, Roche Diabetes Care GmBH, Mannheim, Germany) and analyzed using the Reflotron^®^ system analyzer (F. Hoffmann–La Roche Ltd., Basel, Switzerland). Before sample collection, the puncture site was thoroughly cleaned to remove dirt and sweat. According to Chrismas et al. [26], this method has a high measurement reliability (ICC = 0.90; Typical error of measurement = 94 U/L). The “normal” reference range for CK activity is 24–195 U/L [27].

#### 2.3.6. Well-Being Questionnaire (WBQ)

The WBQ was used in this study, as recommended by Hooper et al. [28] and McLean et al. [29]. According to Thorpe et al. [30], it is a highly sensitive tool for assessing the degree of post-match recovery in football players. This questionnaire evaluated general indicators of player fatigue, sleep quality, overall muscle soreness, stress, and mood on a five-point scale. Values obtained from the questionnaire were added to obtain the WBQ coefficient-Hooper index [31]. The WBQ was administered in the morning at Pre-M, M + 24, M + 48, and M + 72 to standardize the timing of the assessment. Therefore, the WBQ was not administered immediately post-match on the match day, as the Pre-M assessment had already been completed that morning.

#### 2.3.7. Heart Rate and Activity Demands

Heart rate and players’ location were continuously recorded at 1-s intervals during game-based drills using a Polar Team Pro System (Polar Electro, Kempele, Finland), whereby subjects had HR monitors affixed to their chest at the level of the xiphoid process. Heart rate responses were presented relative to each subject’s individualized HRmax, which was determined by laboratory measurement. Raw data from HR monitors were exported into Microsoft Excel (v16.35; Microsoft Corporation; Redmond, WA, USA). To express training impulse, Edwards SHRZ was calculated as SHRZ $=\sum_{i=1}^{5}t_{i}*i$, where *t_i_* was time spent in five heart rate zones: Zone 1 = 50–60% of HRmax, Zone 2 = 60–70% of HRmax, Zone 3 = 70–80% of HRmax, Zone 4 = 80–90% of HRmax, Zone 5 = 90–100% of HRmax [32]. To describe external load, total distance covered (TD), high-intensity distance (HID) as distance covered by speed >14.4 km·h^−1^ [33], and PlayerLoad as PL^TM^ = $\sqrt[{2}]{\frac{{\left(a_{y1}-a_{y-1}\right)}^{2}+{\left(a_{x1}-a_{x-1}\right)}^{2}+{\left(a_{z1}-a_{z-1}\right)}^{2}}{100}}$ were calculated, where *a_y_* was the straight acceleration vector, *a_x_* was the side acceleration vector, and *a_z_* was the vertical acceleration vector.

### 2.4. Statistical Analysis

To determine the minimal number of participants, G*Power (V 3.1, Heinrich-Heine-Universität, Düsseldorf, Germany) with α = 0.05, β = 0.20, and expected effect size 0.8 was used. The minimum sample size required was 20. The a priori sample size calculation was based on the primary repeated-measures analysis and was not designed to determine statistical power for the exploratory correlation analyses. Data analysis was performed using Statistica 14 (Stat Soft, Inc., Palo Alto, CA, USA) with alpha ≤ 0.05. Mean and SD were calculated for each outcome measure. The normality and homogeneity of all data were verified using the Kolmogorov–Smirnov and Levene’s tests. Mauchly’s test indicated that the assumption of sphericity was met for all repeated-measures analyses (all *p* > 0.05), and therefore no correction of the degrees of freedom was required. A repeated-measures ANOVA with one within-subject factor (time of measurement) was used to assess changes in the measured post-match recovery markers over time. When a significant primary effect of the time of measurement was observed, pairwise comparisons were performed using Tukey’s post-hoc test. Partial eta-squared ($\eta_{p}^{2}$) was utilized as a measure of effect size for each analysis of variance, and values were interpreted as small effect (0.01 ≤ 0.06), medium effect (0.06 ≤ $\eta_{p}^{2}\;$< 0.14), and large effect ($\eta_{p}^{2}$ ≥ 0.14). The effect sizes for paired comparisons were expressed by Cohen’s d; d ≤ 0.2 was a small effect and d ≥ 0.8 a large effect. Correlation analyses were considered secondary and exploratory. No adjustment for multiple comparisons was applied; therefore, these findings should be interpreted cautiously and regarded as hypothesis-generating. The criteria adopted to categorize the magnitude of the correlation (r) between the different measures were ≤0.1 as trivial, >0.1–0.3 as small, >0.3–0.5 as moderate, >0.5–0.7 as large, >0.7–0.9 as very large, and >0.9–1.0 as almost perfect [34].

## 3. Results

The workload characteristics of the friendly match and subsequent training sessions are presented in Table 1. Descriptive statistics and changes in fatigue markers across the recovery period are presented in Table 2.

Significant acute post-match changes were observed in several fatigue indicators. Perceived muscle soreness measured using the VAS increased markedly immediately after the match (F = 32.55, *p* < 0.001, =0.71). Similarly, CK concentrations increased significantly following the match (F = 30.16, *p* < 0.001, =0.56).

Neuromuscular performance also showed significant impairments. CMJ height (F = 16.57, *p* < 0.001, =0.35) and reactive strength index (F = 26.19, *p* < 0.001, =0.46) decreased significantly following the match. In addition, sprint performance declined for both the 10-m (F = 32.63, *p* < 0.001, $\eta_{p}^{2}$ = 0.63) and 20-m distances (F = 19.98, *p* < 0.001, =0.39). No significant changes were observed for the 5-m sprint distance (F = 0.51, *p* = 0.39, $\eta_{p}^{2}$ = 0.02).

During the recovery period, several fatigue markers gradually returned to baseline values. Perceived soreness (VAS) remained elevated at 24 h post-match (*p* = 0.041, d = 0.27 after 24 h) but showed a clear reduction by 48 h. Similarly, general muscle soreness assessed via the well-being questionnaire decreased progressively during the 48-h recovery period (*p* = 0.045, d = 0.25 after 24 h), as did stress level (*p* = 0.044, d = 0.25 after 24 h).

CMJ height remained significantly reduced compared with pre-match values at M + 24 (*p* = 0.001, d = 0.99), whereas no significant difference from pre-match was observed at M + 48 (*p* = 0.11, d = 0.28), indicating recovery of CMJ height by 48 h, as well as 20-m sprint performance after 48 h post-match (*p* = 0.001, d = 1.82 after 24 h but *p* = 0.81, d = 0.23 after 48 h). CK concentrations increased significantly during the recovery period, with peak values observed at 24 h post-match (*p* < 0.001). Elevated CK levels persisted at 48 h and remained significantly higher than pre-match values even at 72 h post-match, indicating a prolonged biochemical response following the friendly match (*p* = 0.001, d = 1.55 after 24 h; *p* = 0.001, d = 1.13 after 48 h; *p* = 0.001, d = 1.44 after 72 h).

We found no significant relationship between markers of recovery dynamics and maturity offset, quality of sleep, and hydration among the players. Neither biological age nor hydration status was significantly associated with recovery dynamics.

Significant relationships were observed between several subjective and objective fatigue indicators. At baseline, perceived muscle soreness (VAS) was strongly correlated with CK concentrations (r = 0.59).

Following the friendly match, post-match VAS scores were moderately to strongly associated with CK concentrations measured immediately after the match (r = 0.51), as well as at 24 h (r =0.50) and 72 h post-match (r = 0.52). Post-match VAS scores also showed a large correlation with well-being questionnaire scores measured 24 h after the match (r = 0.51) and were moderately associated with general muscle soreness (r = 0.44) and CMJ height measured 24 h post-match (r = −0.53). In addition, higher CK levels were associated with lower CMJ height immediately post-match (r = −0.61) and 24 h after the match (r = −0.58). During the recovery phase, perceived soreness measured 24 h post-match was significantly associated with CMJ height at 48 h (r = −0.58) and 72 h (r = −0.45).

## 4. Discussion

The present study provides a comprehensive description of recovery pattern differences according to the marker assessed in elite adolescent football players by combining neuromuscular, biochemical, and perceptual measures under standardized match conditions. The friendly match induced substantial fatigue across all monitored domains, although the subsequent recovery pattern differed according to the marker assessed. Functional performance recovered relatively quickly, whereas CK concentrations remained elevated throughout the 72-h observation period. In addition, meaningful associations between subjective and objective measures indicate that different aspects of recovery are closely interconnected.

The reductions observed in CMJ performance and repeated sprint ability immediately after the match are consistent with previous reports identifying these tests as sensitive indicators of neuromuscular fatigue in football players [4,5,35]. The decline in jump height and sprint performance most likely reflects the cumulative effects of repeated accelerations, decelerations, changes of direction, and other eccentric actions performed during match play [6,36]. Such activities place considerable mechanical stress on the neuromuscular system and temporarily reduce the ability to generate force rapidly.

Reactive strength index also declined immediately after the match, indicating an acute impairment of stretch–shortening cycle function. Because RSI reflects the efficiency of force absorption and reutilization, reductions in this parameter may compromise dynamic joint stability during explosive football-specific movements [37]. Similar post-match responses have been reported previously in both youth and adult football players [38,39]. Interestingly, RSI returned to baseline sooner than CMJ height. This faster recovery may reflect the relatively moderate competitive load experienced by youth players together with their greater capacity to restore neuromuscular function after intermittent high-intensity exercise.

Sprint performance followed a recovery pattern comparable to CMJ, with impairments persisting for approximately 24 h before returning to baseline by 48 h. The absence of significant changes in 5-m sprint performance agrees with previous observations suggesting that maximal sprinting ability is more susceptible to match-induced fatigue than initial acceleration [40,41]. Restoration of phosphocreatine availability together with recovery of neuromuscular coordination probably contributed to the relatively rapid normalization of repeated sprint performance [42].

Unlike functional performance measures, CK concentrations remained elevated throughout the entire monitoring period. Peak values occurred 24 h after the match, which is consistent with previous studies reporting post-match elevations in CK concentrations [43,44]. This prolonged response is expected because repeated eccentric contractions during football cause structural disruption of muscle fibers, initiating inflammatory and repair processes that continue even after physical performance has largely recovered [45,46]. The prolonged CK response may appear to contrast with previous reports of faster recovery in youth players. However, our findings suggest that recovery is marker-specific, as functional measures returned to values not significantly different from baseline within 48 h, whereas CK remained elevated throughout 72 h. Thus, faster recovery in youth may be more evident for functional than biochemical markers.

One of the most notable findings of the present study was the discrepancy between functional recovery and the CK response. Although neuromuscular performance had largely returned to baseline within 48 h, CK concentrations remained significantly elevated throughout the 72-h observation period. This finding demonstrates that recovery trajectories differ depending on the marker assessed. However, because CK was the only biochemical marker measured, its persistent elevation should not be interpreted as direct evidence of ongoing structural muscle damage. Rather, CK provides complementary information about the prolonged biochemical response to match play [2,47,48]. Consequently, practitioners should avoid relying solely on performance tests when evaluating recovery status after match play.

Subjective measures showed a recovery profile that complemented the objective assessments. Muscle soreness remained elevated 24 h after the match despite improvements in several performance variables, which is consistent with the delayed inflammatory response commonly associated with eccentric exercise [25,49,50]. However, the association between VAS and CK already observed at Pre-M may reflect residual fatigue or muscle stress from the preceding training rather than a match-related response. Furthermore, the observed correlations between VAS scores, CK concentrations, and CMJ performance support previous evidence indicating that athletes’ perceptions provide meaningful information about physiological recovery [30,51]. Because these questionnaires are inexpensive, non-invasive, and easy to administer, they represent a practical addition to routine monitoring programs in elite youth football.

Taken together, the present findings reinforce the value of a multidimensional monitoring approach. Neuromuscular tests such as CMJ and RSA appear particularly useful for tracking short-term functional recovery during the first two days after competition, whereas subjective measures offer additional insight into the player’s perceived readiness. CK measurements provide complementary information regarding the biochemical response to match play but should not be interpreted as a direct measure of readiness to train. Instead, persistently elevated CK concentrations may signal continuing tissue repair despite apparent restoration of physical performance, particularly during congested competition schedules.

Several limitations should be acknowledged when interpreting these findings. Although maturity offset was estimated, biological maturation itself was not directly assessed and may have contributed to individual differences in recovery. Likewise, nutritional intake and sleep were not experimentally controlled and therefore could have influenced post-match responses. The study also focused exclusively on CK as a biochemical marker of muscle damage. Future investigations incorporating inflammatory markers or additional indicators of muscle damage may provide a more complete understanding of recovery processes. Finally, the observation period was limited to 72 h; extending follow-up beyond this timeframe would help determine when biochemical recovery is fully completed. The correlation analyses were exploratory and were not adjusted for multiple comparisons, which increases the possibility of Type I error. In addition, the a priori sample-size calculation was based on the primary repeated-measures analysis rather than the correlation analyses; therefore, the study may have been too underpowered to detect small-to-moderate associations. Although the participating teams were of comparable competitive and performance level and followed the same study protocol, potential between-match or between-team differences were not explicitly accounted for in the statistical analysis.

## 5. Conclusions

The findings indicate that match play induced substantial neuromuscular, biochemical, and perceptual fatigue. Neuromuscular performance and repeated sprint ability showed significant impairments immediately after the match but largely recovered within 48 h. In contrast, creatine kinase concentrations remained elevated throughout the 72-h recovery period, demonstrating that the time course of the CK response differed from that of functional performance markers. Perceptual measures of muscle soreness were moderately associated with both neuromuscular performance and biochemical markers, suggesting that subjective monitoring tools may provide meaningful information about players’ recovery status. These results highlight the importance of using a multidimensional monitoring approach when assessing post-match recovery in elite youth football players. In practical settings, neuromuscular tests and simple perceptual measures may provide useful information for monitoring post-match recovery. However, recovery of these markers should not be considered equivalent to readiness to resume training or competition.

## Figures and Tables

**Table 1:** Workload Description of Training Sessions.

	TD (m)	HiD (m)	ACC	PL^TM^ (a.u.)	SHRZ (a.u.)
Match	9876 ± 725	181 ± 29	87 ± 9	512 ± 43	445 ± 23
Match + 24 h	3573 ± 384	9 ± 1	31 ± 8	212 ± 36	111 ± 26
Match + 48 h	5013 ± 299	77 ± 12	52 ± 11	121 ± 14	171 ± 17

Table notes: TD—total distance covered, HiD—high-intensity distance covered (>14.4 km·h^−1^), PLTM—player load, SHRZ—summated heart rate zones.

**Table 2:** Changes in Neuromuscular, Biochemical, and Perceptual Recovery Markers Following the Match.

	Post-Match Fatigue		Recovery Dynamics		
	Pre-Match	Post-Match	M + 24	M + 48	M + 72
VAS (mm)	12.63 ± 6.73	78.97 ± 13.06 ^#^	27.03 ± 11.98 ^#^	16.59 ± 8.14	18.34 ± 10.11
CK (U/L)	187.46 ± 41.14	362.17 ± 169.84 ^#^	389.53 ± 183.89 ^#^	364.47 ± 211.75 ^#^	389.31 ± 161.83 ^#^
CMJ_h_ (cm)	30.75 ± 4.13 ^#,§^	27.14 ± 3.41 ^#^	27.89 ± 2.31 ^#^	29.13 ± 3.15 ^§^	29.88 ± 2.57 ^§^
CMJ_RSI_	1.18 ± 0.31	0.81 ± 0.26 ^#^	1.08 ± 0.27	1.13 ± 0.27	1.09 ± 0.29
TT 5 m (s)	4.96 ± 0.56	5.22 ± 0.61	5.07 ± 0.59	4.91 ± 0.33	4.96 ± 0.21
TT10 m (s)	9.17 ± 0.53	12.29 ± 0.83 ^#^	11.69 ± 0.65 ^#^	9.28 ± 0.57 ^§^	9.08 ± 1.75 ^§^
TT20 m (s)	15.75 ± 0.79	18.24 ± 3.53 ^#^	18.30 ± 0.80 ^#^	15.33 ± 3.44 ^§^	15.43 ± 3.12 ^§^
Fatigue	2.00 ± 0.81	NA	3.03 ± 0.82	2.62 ± 0.79	2.87 ± 0.87
Sleep	2.38 ± 0.60	NA	2.18 ± 0.53	2.25 ± 0.56	2.18 ± 0.73
Muscle soreness	2.62 ± 0.44	NA	3.51 ± 0.82 ^#^	2.75 ± 0.76	2.75 ± 0.72
Stress level	2.12 ± 0.47	NA	2.78 ± 0.29 ^#^	1.93 ± 0.84	2.12 ± 0.70
Mood	1.87 ± 0.70	NA	1.93 ± 0.43	2.00 ± 0.62	1.81 ± 0.53
Hooper Index	11.01 ± 2.78	NA	12.15 ± 1.81	11.56 ± 2.66	11.75 ± 2.44
∆BM (%)	NA	1.67 ± 0.13	1.07 ± 0.16	0.63 ± 0.09 ^§^	0.31 ± 0.07 ^§^

Table notes: ^#^—significantly different from pre-match values (*p* ≤ 0.05), ^§^—significantly different from M + 24 values, NA indicates that the WBQ was not administered immediately post-match, as all WBQ assessments were standardized to morning measurements, and similar ∆BM (%) was administered post-match.

## Data Availability

The data presented in this study are available on reasonable request from the corresponding author.

## Abbreviations

The following abbreviations are used in this manuscript:

CK	Creatine kinase
VAS	Visual analog scale
CMJ	Counter movement jump
WBQ	Well-being questionnaire
HC	Hydration control
RSA	Repeated sprint ability test
